# Age Evolution of Lipid Accretion Rate in Boars Selected for Lean Meat and Duroc Barrows

**DOI:** 10.3390/ani12141868

**Published:** 2022-07-21

**Authors:** Laura Sarri, Joaquim Balcells, Ahmad Reza Seradj, Ramona N. Pena, Gustavo A. Ramírez, Marc Tor, Gabriel de la Fuente

**Affiliations:** Departament de Ciència Animal, CERCA Center, Universitat de Lleida-Agrotecnio, Av. Alcalde Rovira Roure 191, 25198 Lleida, Spain; laura.sarri@udl.cat (L.S.); joaquim.balcells@udl.cat (J.B.); reza.seradj@udl.cat (A.R.S.); romi.pena@udl.cat (R.N.P.); gustavo.ramirez@udl.cat (G.A.R.); gabriel.delafuente@udl.cat (G.d.l.F.)

**Keywords:** apparent digestibility, incorporation rate, pigs, producing type, stearoyl-CoA desaturase, unsaturation rate

## Abstract

**Simple Summary:**

Fat deposition profile and fatty acid biosynthesis in adipose tissues are two relevant metabolic processes for breeding companies as the fat content of meat relates to its technological, organoleptic, and nutritional quality. Age, breed, and sex have a great impact on fat metabolism. Therefore, in the present experiment, a labeled saturated fatty acid was added to the diet of pigs belonging to three different producing types during two growth phases, to define the rate of its incorporation into body fat. In addition, the activity of the enzyme stearoyl-CoA desaturase, which promotes the conversion of some saturated fatty acids into monounsaturated, was analyzed. In the growing phase, direct incorporation of fat from diet was higher than biosynthesis in most adipose tissues, whereas in the fattening phase it was higher only in the liver, revealing the changing roles of organs in fat metabolism with age. The leaner pigs also obtained a higher fat incorporation, denoting higher reliance on dietary fatty acids for fat deposition; overall, real stearoyl-CoA desaturase activity was higher in the fattening phase.

**Abstract:**

Fatty acid (FA) deposition in growing–fattening pigs is mainly based on endogenous lipid synthesis, but also direct FA incorporation from the diet. To evaluate the direct fat incorporation rates and the endogenous desaturation action of the stearoyl-CoA desaturase (SCD) enzyme, a deuterium (D)-labeled saturated FA (d_35_-C18:0) was added to the diet. Sixteen three-way (3W) crossbred boars, and thirty-two purebred Duroc barrows homozygous for the *SCD* single nucleotide polymorphism rs80912566 (16 CC/16 TT), were used. Half of the animals of each genotype belonged to the growing and fattening phases. The fractional incorporation rate (FIR) of dietary fat in growing pigs was generally higher in adipose tissues, whereas in fattening pigs it was higher in the liver. Duroc pigs exhibited lower FIRs than 3W pigs, suggesting lower rates of endogenous synthesis by 3W pigs. Real fractional unsaturation rates (FURs) increased with age by the higher FIRs in 3W pigs and the de novo synthesis pathway in Duroc genotypes. Moreover, pigs carrying the *SCD*_T allele showed more enhanced oleic acid biosynthesis than Duroc CC pigs. In conclusion, suitable feeding protocols should be designed for each pig type to optimize production traits, considering that the metabolic pathway of FA for its deposition may differ.

## 1. Introduction

Swine selection has traditionally focused on lean productive efficiency; however, consumer demand for high-quality products has considerably increased over the last years [1]; therefore, traditional fatty breeds such as Iberian or specific Duroc lines have been selected for relevant quality traits such as higher levels of intramuscular fat (IMF) [2,3]. To enhance these quality traits, pigs are raised to a heavier slaughter weight and males are usually castrated to avoid boar taint, as they reach sexual maturity [4,5]. The Duroc breed has long been selected for lean growth, although certain lines have preserved and expressed both high lipogenic activity and lipid deposition capacity [6]. In these fattier producing types (PTs), the most sought-after effects are increased IMF content and improved fatty acid (FA) composition [7,8], by the promotion of monounsaturated FA in detriment of saturated FA, bearing in mind the reported adverse effects of saturated FA on human health [9,10].

Despite the use of suitable genetic lines to obtain premium meat products, the management of fat incorporation throughout the growing–fattening phases is complex, as it involves the deposition of FA into adipocytes from both sources, diet and endogenous origins. In pigs, lipid synthesis occurs mostly in adipose tissue [11], and it is generally accepted that deposited lipids from lipid synthesis exceed those from direct dietary incorporation, with endogenous oleic (C18:1 *c*9), palmitic (C16:0), and stearic (C18:0) acids accounting for 80% of total FA deposited [12,13]. Thus, FA composition in tissues is related to specific rates of deposition, synthesis, and desaturation [14]. Transformation of saturated FA to monounsaturated FA is enhanced by a single nucleotide polymorphism of the stearoyl CoA desaturase gene (*SCD*; rs80912566), which regulates a rate-limiting enzyme (SCD) that has a main function of maintaining proper fluidity of cellular lipids without affecting IMF content or backfat thickness. The main substrates of SCD are palmitoyl and stearoyl-CoA, which are desaturated at the Δ9 position, transforming them into de novo palmitoleoyl and oleoyl-CoA, respectively. The *SCD*_T allele enhances this process compared with the alternative *SCD*_C allele [15], thus promoting the synthesis of monounsaturated FA.

To optimize fat deposition and enable a comprehensive understanding, it is necessary to build a dynamic model of lipid development that combines endogenous FA de novo synthesis and exogenous dietary deposition [16,17]. In this context, the biochemical and genetic mechanisms involved in fat deposition between fatty and lean PTs needs to be clarified [14]. Therefore, the objective of the present study is to evaluate lipid metabolism in different pig PTs by analyzing both de novo synthesis and dietary FA incorporation into several tissues.

## 2. Materials and Methods

Protocols and experimental procedures were approved by the Ethics Committee for Animal Experiments of the University of Lleida (Ref: CEEA 09–05/16). The care and use of animals were in accordance with the Spanish Policy for Animal Protection RD 53/2013, which meets the European Union Directive 2010/63 on the protection of animals used for experimental purposes.

### 2.1. Animals, Diets, and Experimental Design

The experiment was carried out in the Swine Research Centre (CEP; Torrelameu, Lleida, Spain) using 48 male pigs at two growth phases (GP), 24 growing pigs (28.42 ± 0.861 kg of body weight (BW); µ ± SE) and 24 fattening pigs (87.40 ± 1.256 kg BW). Each GP was composed of three PTs of eight pigs each. The lean type consisted of (i) 8 three-way (3W) crossbred boars [Pietrain sires × (Duroc × Landrace) dams], while the fatty type consisted of 16 purebred Duroc barrows, of which (ii) 8 were homozygous for the *SCD*_C allele, and the remaining (iii) 8 were homozygous for the *SCD*_T allele for the *SCD* rs80912566 genotype. Pigs were genotyped by allelic discrimination assay with primers and probes following Estany et al. [15]. Upon arrival at the experimental facility, pigs within the same GP and PT were randomly assigned to one of the two dietary treatments and placed in groups of four in 55% concrete slatted-floor pens (2.10 × 2 m^2^), where they remained the first 5 days as a dietary adaptation period. During the last 6 days, pigs were housed individually in metabolic cages (2 × 1.04 m^2^), weighting the pigs just before housing them in the metabolic cages. For each GP, two experimental diets [standard (SP) and low (LP) protein] were formulated as previously described [18]. Titanium dioxide (TiO_2_; 5 g/kg dry matter (DM)) was included as an inert flow marker, and 290 mg/kg DM of deuterium (D)-labeled stearic acid (d_35_-C18:0; Tracer, Madrid, Spain) was also included in each experimental diet on the last 6 days of each GP. Diets were supplied ad libitum, measuring the daily feed supply and waste, and pigs had free access to drinking water. The ingredients and chemical composition of the experimental diets can be reviewed in a previous study [18], while the dietary FA composition is shown in Table 1. Pigs were culled on day 11.

### 2.2. Sample Collection and Dissection Process

Blood samples were collected daily (between 0.900 and 1000 h) without fasting in ethylenediamine tetraacetic acid tubes from the jugular vein for five consecutive days before culling. Plasma was obtained by centrifugation (2500 rpm for 10 min) and stored at 80 °C for further analysis. On the last day, animals were euthanized by an intravenous infusion of sodium thiopental (Esteve S.A., Oudewater, The Netherlands), and immediately weighted and eviscerated. Age at slaughter was 79.43 ± 1.875 and 159.38 ± 3.359 days of age for 3W pigs, and 86.25 ± 1.356 and 150.69 ± 2.323 days of age for Duroc pigs for the growing and fattening phases, respectively. Digesta from the last part of the upper intestine (15–20 cm) was taken to determine apparent ileal digestibility (AID); and subcutaneous adipose tissue (SC), liver, and longissimus dorsi and semimembranosus skeletal muscles were sampled from the left half carcass. Samples of SC and longissimus dorsi muscle were taken between the third and fourth last ribs, while those of semimembranosus muscle were taken from the third most proximal to the spine. Samples were stored immediately at −80 °C until required for fat and FA determinations. The loin and leg from the right half carcass were cut based on Walstra and Merkus [19] standards, and reserved for relative allometric coefficients (k) determination by dissecting tissues. The liver and the right half carcass loin were weighted, as well as the right leg, which was dissected into muscles, bones, skin and SC, intermuscular tissue, and remainder (blood vessels, ligaments, and tendons) in a controlled-environment dissection room. Measurements of IMF were performed in gluteus medius and semimembranosus muscles by the Sohxlet method according to the Association of Official Analytical Chemists [20] (ref. 920.39).

### 2.3. Analytical Procedures

#### 2.3.1. Apparent Ileal Digestibility

Samples of ileum content were freeze-dried and homogenized and, along with experimental diet samples, were analyzed for DM content [20] (ref. 934.01). Crude protein (CP) content (nitrogen × 6.25) was analyzed by Dumas combustion (Tru Spec CN; Leco Corporation, St. Joseph, MI, USA) [21]. Fatty acid methyl esters were obtained in duplicate by transesterification of 75 mg-samples [22]. Titanium dioxide was analyzed in ileum and dietary ashes using inductively coupled plasma mass spectroscopy (7700×, Agilent Technologies, Tokyo, Japan) following the Darambazar [23] procedure with some modifications, including the digestion with 6.5 mL H_2_SO_4_ (7.4 M) for 1.5 h at 200 °C and the addition of 5 mL H_2_O_2_ (30%, *v*/*v*) after cooling.

#### 2.3.2. Tissue Fatty Acids Analysis

Total lipids from plasma, liver, and adipose tissues (including SC and IMF of longissimus dorsi and semimembranosus muscles) were extracted [24] using a chloroform-methanol solution (2:1, *v*/*v*). Solvents were evaporated under vacuum (at 40 °C), and FA were subsequently obtained by saponification [25] by adding 1.2 mL of saponification solution [KOH (5M) in methanol/water (50:50, *v*/*v*)], and placing the sealed tubes, flushed with nitrogen, in a water bath during 60 min at 60 °C. Then, glacial acetic acid was added to neutralize the KOH fraction and FA were extracted using petroleum spirit. The solvent was evaporated under vacuum and samples were re-diluted in isopropanol, vortexed, and filtered through a 0.2 µm hydrophilic polytetrafluoroethylene membrane.

Measurements of D-labeled FA were performed in duplicate by ultra-high-performance liquid chromatography coupled to a Xevo triple quadrupole mass spectrometer (TQD; Waters, Milford, MA, USA). The system was equipped with an electrospray ionization source and an ACQUITY HSS-T3 column (2.1 × 150 mm; 1.8 µm). A multiple reaction monitoring method was designed and optimized to include the following FA: C18:0, d_35_-C18:0, C18:1 *c*9, and d_33_-C18:1 *c*9. Cone voltage and collision energy were optimized for all transitions, and the absence of cross-signaling between D-labeled and unlabeled FA channels was checked. Results were processed using QuanLynx V4.1 software (MassLynx, Waters Corporation, Milford, MA, USA).

### 2.4. Calculations

Nutrient AID was calculated as described in Sarri et al. [18] as follows:

$$y=1\;–\;(\;\frac{{\mathrm{marker}}_{\mathrm{feed}}}{{\mathrm{marker}}_{\mathrm{ds}}}\;\times \;\frac{{Ζ}_{\mathrm{ds}}}{{Ζ}_{\mathrm{feed}}}\;)$$

where y is the coefficient of AID of a nutrient; Ζ_ds_ and Ζ_feed_ are the nutrient concentration in ileum and in the diet, respectively; and marker _feed_ and marker _ds_ represent marker (TiO_2_) concentrations in diet and ileum, respectively.

Plasma and tissue d_35_-C18:0 enrichment was expressed as molar percent excess (MPE) [18]. The percentage of plasma d_35_-C18:0 incorporated per day (fractional incorporation rate: FIR, %/day) into fat depots was calculated from the equation:

$$\mathrm{FIR}=(\;\frac{{\mathrm{MPE}\;d}_{35}{–\;C18:0}_{\mathrm{tissue}}}{{\mathrm{aveMPE}\;d}_{35}{–C18:0}_{\mathrm{plasma}}}\;\times \;\frac{100}{t}\;)$$

where MPE d_35_-C18:0_tissue_ is the enrichment in d_35_-C18:0 of stearic acid in tissues; aveMPE d_35_-C18:0_plasma_ is the average d_35_-C18:0 enrichment of stearic acid in the plasma pool during the labeled diet consumption; and t is the labeling time in days.

Apparent and real fractional unsaturation rates (FURs) in SC were calculated as the percentage of C18:0 and d_35_-C18:0 unsaturated per day (%/day) to C18:1 *c*9 and d_33_-C18:1 *c*9, respectively.

Both were calculated as follows:

$$\mathrm{Apparent}\;\mathrm{FUR}=(\;\frac{{C18:1\;c9}_{\mathrm{tissue}}}{{C18:0}_{\mathrm{tissue}}}\;\times \;\frac{100}{t}\;)$$

$$\mathrm{Real}\;\mathrm{FUR}=(\;\frac{d_{33}{–C18:1\;c9}_{\mathrm{tissue}}}{d_{35}{–C18:0}_{\mathrm{tissue}}}\;\times \;\frac{100}{t}\;)$$

where C18:1 *c*9_tissue_ and C18:0_tissue_ of apparent FUR are the enrichment in C18:1 *c*9 and C18:0 FA in the same tissue, respectively; both FA in this index can come from endogenous and exogenous sources; while in the real FUR, d_33_-C18:1 *c*9_tissue_ and d_35_-C18:0_tissue_ are the enrichment in d_33_-C18:1 *c*9 of oleic acid and d_35_-C18:0 of stearic acid in the same tissue, respectively, and should reflect SCD activity; and t is the labeling time in days.

The relative growth coefficients (*k*) of each dissection component of the right leg were studied in relation with body growth. They were obtained as the slope of the regression of each component weight on body weight from the allometric equation (*y* = *a*$x^{k})$ [26], with both logs transformed to linearize the equation as follows:

log (*y*) = *k* log (*x*)–log (*a*)

where log *y* is the weight of each leg component, log *x* is the body weight, log *a* is the intercept, and *k* is the allometric growth coefficient.

### 2.5. Statistical Analysis

The statistical analysis of AID, FIR, and apparent and real FUR was performed by applying a MIXED model using SAS statistical software (v9.4; SAS Institute Inc., Cary, NC, USA), including the GP (growing and fattening) and PT (Duroc CC, Duroc TT, and 3W) and their two-way interaction as fixed effects, where each pig was considered an experimental unit. Because moderate restriction of dietary CP over such a short period of time did not imply significant differences or interactions in any of the parameters studied, the effect was dropped from the model for these traits. To consider the repeated measures (GP), the residual was modeled using an unstructured covariance matrix. Differences between least square means were assessed using the Student’s *t*-test. Results were reported as least square means and their SE. Significant differences and tendencies were declared at *p* ≤ 0.05 and 0.05 < *p* < 0.10, respectively.

The allometric coefficients for the liver, loin, and different leg components and their standard errors were estimated using overall data (growing and fattening phases) and, within PTs, using generalized linear model (GLM) procedures. The Student’s *t*-test was used to test if each coefficient was statistically different from 1.

## 3. Results

### 3.1. Production Data and Allometric Tissue Growth

A summary of intakes and performance parameters is provided in Table 2. Pigs significantly increased voluntary feed intake with age (*p* < 0.001). Although no differences were found between PTs in the growing phase, in the fattening phase Duroc CC pigs showed significantly higher feed intake than Duroc TT pigs (GP × PT interaction effect; *p* = 0.032). In terms of production performance, fattening pigs also showed higher average daily gain (ADG) than growing pigs (*p* < 0.001). No differences between PTs were found in growing pigs, whereas in the fattening phase 3W pigs had higher ADG than Duroc TT pigs, and Duroc CC pigs were in between 3W and Duroc TT pigs (GP × PT interaction effect; *p* = 0.045). The same was observed with the feed:gain ratio, although no significant interaction between GP and PT was detected.

To assess the maturity kinetics between PTs in various organs and carcass components, average allometry coefficients at 80 and 150 days of age (59 kg difference in BW) were calculated and are shown in Table 3.

Liver and bones showed allometry coefficients below 1, indicating an advanced degree of maturity as corresponds to vital organs and the skeletal system. However, the opposite was seen in SC, intermuscular fat, and IMF of gluteus medius, showing allometry coefficients up to 1, indicating a high rate of relative growth. The intermuscular adipose tissue of Duroc pigs was an exception, as its allometric coefficient was not dissimilar to 1, and was lower than that of the leaner type 3W pigs (*p* = 0.015). It should be noted that IMF of semimembranosus, as well as the semimembranosus muscle itself, showed a relative growth coefficient lower than 1 in both PTs, suggesting a higher degree of maturity of this muscle. Moreover, Duroc pigs presented a ham muscle allometry coefficient higher than 1, whereas in 3W pigs the ham allometry coefficient did not differ from 1.

### 3.2. Apparent Ileal Digestibility

The AID coefficients of DM, CP, and EE are shown in Table 4. The effect of PT on AID interacted with GP (GP × PT interaction effect). In growing phase, 3W pigs showed lower AID than Duroc pigs for DM, CP, and EE, while AID did not differ between Duroc genotypes (CC and TT). However, in the fattening phase there were no differences in AID among PTs. Only in 3W pigs did AID of CP and EE increase with age (*p* < 0.05).

**Figure 1:**
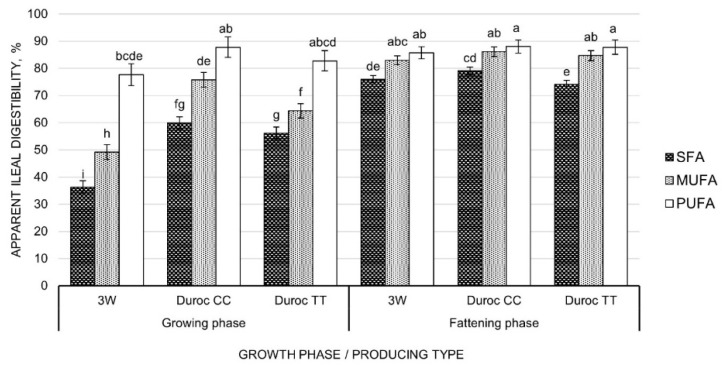
Apparent ileal digestibility of fatty acids, by three-way (3W) crossbred pigs and Duroc purebred pigs of TT/CC *SCD* genotype, at two growth phases (growing vs. fattening). ^a, b, c, d, e, f, g^ means with different superscripts differ significantly (*p* ≤ 0.05). Saturated fatty acids (SFA): C14:0, C16:0, C17:0, C18:0, C20:0, and C22:0; Monounsaturated fatty acids (MUFA): C16:1, C18:1 *c*9, C18:1 *c*11, and C20:1 *c*11; Polyunsaturated fatty acids (PUFA): C18:2 *c*9, *c*12 and C18:3 *c*9, *c*12, and *c*15.

The AID of saturated, monounsaturated, and polyunsaturated FA is reported for each GP and PT in Figure 1. Polyunsaturated FA showed the highest AID, reaching values around 80%, with no significant differences between the three PT within each GP. Monounsaturated FA were less digested than polyunsaturated FA in the growing phase, but differences disappeared with animal development. In the growing phase, the AID of monounsaturated FA differed among PT, being better digested in Duroc CC, followed by Duroc TT and finally by 3W pigs. Saturated FA showed the lowest AID in both GP, although it increased significantly in the fattening phase. While growing 3W pigs had the lowest AID of saturated FA, reaching close to 40%, in the fattening phase 3W pigs reached both Duroc genotypes, and Duroc CC showed significantly higher AID than Duroc TT pigs.

### 3.3. Fractional Incorporation Rate (FIR)

The FIR of dietary stearic acid (d_35_-C18:0) is an index of daily incorporation of FA relative to its plasma availability. Estimated values of FIR in the liver, SC, longissimus dorsi, and semimembranosus IMF are shown in Table 5. The FIR differed significantly among the tissues studied; in the liver, fattening pigs showed a 3.4-fold higher FIR than growing pigs (*p* < 0.001), with no interaction with PT. However, in the SC the GP and PT did interact (*p* = 0.037), so that significant differences were observed among PT in fattening but not in growing pigs. The 3W pigs significantly increased their SC-FIR over time, whereas in Duroc TT and Duroc CC it remained stable or numerically decreased in Duroc CC pigs. Moreover, the FIR of the longissimus dorsi muscle was impacted by both PT and GP. Regarding GP, the FIR of the longissimus dorsi muscle was much higher in the growing than in the fattening phase (*p* < 0.001); this effect was true in all PT, although it was more pronounced in 3W animals (GP × PT interaction effect; *p* = 0.051). In semimembranosus muscle, FIR decreased in Duroc CC and 3W pigs in the fattening phase, whereas it remained stable in Duroc TT pigs. In 3W pigs, the dietary d_35_-C18:0 FA in semimembranosus muscle was under the limit of quantification.

### 3.4. Oleic Acid De Novo Synthesis: Δ9-Desaturase Activity

Oleic acid in fat depot comes from two sources: oleic acid synthesis as a product of Δ9-desaturase activity and direct incorporation of dietary oleic acid. Apparent Δ9-desaturase activity (Table 6) is commonly determined through the FA profile of adipose tissue. Indeed, this value represents the result of both anabolic and catabolic lipid processes occurring throughout the lifespan of the animal and does not reflect necessarily the activity at a specific time-interval. Apparent Δ9-desaturase activity is also masked by oleic acid deposited directly from the diet.

To evaluate the real Δ9-desaturase activity at a specific time-interval, the appearance of d_33_-C18:1 *c*9 FA in SC originated from the administered form of D-labeled stearic acid (d_35_-C18:0) was quantified. Table 6 shows the estimated apparent and real FUR as a measure of the oleic acid synthesis. Thus, the apparent activity corresponds to the entire lifespan of the pig, whereas the real activity corresponds to the specific oleic acid synthesis (or FUR) that happened during the specific 6 day-period of the study. The PT and GP affected apparent FUR and showed a significant interaction between them (*p* < 0.001). Apparent FUR was higher in growing than in fattening phase (*p* < 0.001). Growing 3W pigs showed the highest FUR values, followed by Duroc TT and Duroc CC pigs. However, in the fattening phase, apparent FUR values in 3W and Duroc TT pigs were similar, while Duroc CC pigs kept a significantly lower FUR value. Regarding real FUR activity, it behaved opposite to the apparent FUR as pigs aged; thus, fattening animals presented significantly greater FUR values (*p* < 0.001) than growing animals. In addition, significant differences between PTs were also detected but only in the fattening phase (GP × PT interaction effect; *p* = 0.016), where 3W and Duroc TT pigs had higher FUR values than Duroc CC pigs.

## 4. Discussion

The authors are aware that the number of individuals per group in the present trial may limit the accuracy of the data; however, the same individuals were simultaneously used to analyze the kinetics of protein synthesis to obtain a complete picture of nutrient metabolism in pigs [18]. The whole process required surgical catheterization and confinement of pigs in metabolic cages; therefore, the experimental design restricted the available number of experimental animals.

This study aimed to compare specific aspects of the fat metabolism of two types of pigs generally used in southern Europe for different productive purposes. The 3W crossbred pigs come from intensive selection to enhance lean carcass and feed conversion; these leaner pigs are slaughtered at lighter weights to take advantage of their high growth potential. However, heavy pigs intended for high-quality pork (e.g., specific Duroc lines or Iberian pigs) are slaughtered at heavier weights and males are usually castrated to avoid boar taint and improve fat deposition [27]. Thus, the term PT used throughout the manuscript is employed because it includes both genotype and castration traits, as Duroc barrows were surgically castrated shortly after birth to meet commercial conditions. In addition, within the heavy Duroc line, pigs were genotyped and selected to be homozygous for the *SCD*_T and *SCD*_C alleles to exploit their differential Δ9-desaturase activity and oleic acid synthesis capability [15]. This study was performed in two GP (28.42 ± 0.861 kg BW and 87.40 ± 1.256 kg BW for growing and fattening phases, respectively), in which growing pigs showed differential rates of protein synthesis [18] and also of fat metabolism, with a subsequent accretion that may be altered by selection strategies exerted on the genotypes used.

Fatty acids are deposited in adipose tissues through two mechanisms: direct FA incorporation, mostly from the diet [28], but also from mobilization from other fat depots; and through de novo synthesis processes, using different precursors at different rates. The biological diversity of FA in adipose tissues and the variety of precursors make fat accretion a complex process. To address this topic, the authors administered D-labeled stearic acid (d_35_-C18:0), with molecular hydrogens of the hydrocarbon chain that were labeled with deuterium, to monitor direct FA incorporation. Likewise, labeled oleic acid (d_33_-C18:1 *c*9), with all hydrogens of the hydrocarbon chain D-labeled, was considered a valid index of endogenous FA de novo synthesis, since it derives directly from the desaturation of dietary d_35_-C18:0 by the action of SCD.

The validity of the FIR relies on the rate between d_35_-C18:0 incorporation into adipose tissues and available d_35_-C18:0 in plasma. Thus, the robustness of the FIR depends on (i) the stability of plasma d_35_-C18:0 enrichment and (ii) the absence of d_35_-C18:0 recycling. Regarding the former point (i), plasma d_35_-C18:0 enrichment leveled off after 72 h of its dietary administration, and consistent detection was obtained in adipose tissues 72 h thereafter. Considering that pigs were fed ad libitum and that digestion and intestinal absorption may buffer the discontinuity of discrete meals, plasma d_35_-C18:0 enrichment was arguably constant. In relation to the last point (ii), the analytical protocol allowed the identification of d_35_-C18:0 in plasma and tissues, so the possibility of background contamination or endogenous return in plasma was negligible. Following the same principle, the appearance of d_33_-C18:1 *c*9 in tissues should be associated with d_35_-C18:0 availability, without any possible interference with exogenous sources. Nevertheless, d_33_-C18:1 *c*9 was only consistently detected in SC with our analytical approach, since SCD activity is significantly higher in this tissue [29,30].

### 4.1. Production Data and Differential Growth Intensity

In line with previous findings [31], Duroc pigs homozygous for the *SCD*_T allele presented lower feed consumption than Durocs carrying the *SCD*_C allele, and tended to show lower performance in the fattening phase.

Regarding allometric coefficients, no abnormalities were observed in the growth profile during the experimental period [32]. Considering that *SCD* genotype variation only affects FA composition but not fat content [15,33], datasets from both Duroc genotypes (TT and CC) were pooled and used as a single group. As previously described [34], pigs showed a high degree of maturity in the liver and a proximal-distal gradient in the hindlimb bones, all in negative allometry. Adipose tissue accretion was in positive allometry, whereas intermuscular fat differed between PTs. These differences in fat metabolism could be attributed to divergence maturity of adipose tissue between fatty and lean pigs [14,34]. The 3W pigs had a higher allometry coefficient in intermuscular fat, and a high tendency in semimembranosus muscle IMF. These results are consistent with an elevated ratio of intermuscular to SC fat, particularly in the Pietrain breed and to a lesser extent in purebred or crossbred Duroc, as well as a higher ratio in boars than in barrows [32].

For skeletal muscles, the semimembranosus was in negative allometry, indicating a higher degree of maturity than other muscles in the ham. Likewise, the allometric growth rate of this muscle indicates how a greater degree of maturity may advance IMF metabolism, which is the last fat depot to develop [35].

### 4.2. Apparent Ileal Digestibility

The results showed a clear effect of GP on the mechanisms regulating fat absorption and deposition; moreover, the effect of GP was not homogeneous and interacted with PT. The AID of EE was higher and homogenous in fattening pigs, whereas in growing pigs EE-AID was impacted by PT, being significantly reduced in 3W pigs. Such interaction also was observed in several FA fractions.

The link between animal development and the rate of FA digestibility was previously suggested by Powles et al. [36], who revealed that the physicochemical structure of FA effectively alters the digestibility process. Digestibility is favored by the increase of the unsaturation rate and the reduction of the hydrocarbon chain length through micelle formation [37,38], although such effect (saturated/unsaturated FA ratio) was found more pronounced in young than in old pigs [36]. Our findings also confirmed that saturated FA were the least digested, followed by monounsaturated and polyunsaturated FA, with mean AID coefficients of 63.59%, 73.87%, and 84.95%, respectively.

It should also be pointed out that dietary EE content and FA profiles differed between diets, with fattening diets containing a higher proportion of EE (60%) than the growing ones. In this regard, previous studies suggested that fat AID may be increased with fat inclusion level [39] or may differ with FA composition [38]. In any case, a differential dietary composition in EE may have masked the effect of age on fat digestibility.

Regarding pig PT, growing 3W pigs showed lower AID for DM, CP, and EE, coinciding with lower rates of AID in saturated and monounsaturated FA, although these coefficients converged in the fattening phase. These findings agree with previous studies [40,41] in which fatty genotypes appeared to use dietary nutrients more efficiently at earlier ages than leaner ones, due to both earlier development of their digestive tract and higher enzyme activity.

### 4.3. Fractional Incorporation Rate of Stearic Acid

Incorporation of dietary fat into tissues differed between liver and adipose tissues, and semimembranosus IMF was the depot with the lowest FIR, as corresponds to a mature organ with a low allometry coefficient. The liver was much more active during the fattening phase in all PTs, whereas IMF of skeletal muscles had higher FIR in the growing phase, showing PT-dependence for each GP. Distinct tissues play different roles in fat metabolism [11,42]; while in pigs adipose tissue is the most active for FA synthesis [11], the liver is mainly involved in lipid oxidation and long-chain PUFA synthesis. Age also affects lipid metabolism on a large scale. Duran-Montgé et al. [42] established that lipogenic gene expression was higher in adipose tissue at 60 kg BW, whereas at 100 kg BW it was greater in the liver. In this regard, it was reported that adipose tissue reached the highest lipogenic activity at 120 days of age, decreasing gradually thereafter [43]. Therefore, both liver and adipose tissue may change their role in fat metabolism over time.

Differences between PTs in SC-FIR showed up mainly in the fattening phase, when fat deposition is predominant [32]. While Duroc pigs maintained their FIR stable throughout age, 3W pigs significantly increased it, along with an improvement in their FA-AID, which suggests an increase of dietary fat incorporation with age. However, in IMF, the FIR of 3W pigs showed a different development than in SC. The longissimus dorsi FIR was higher in 3W than in Duroc pigs at both GP, but experienced the greatest decrease in the fattening phase. This decrease in FIR could explain the lower IMF content in this leaner genotype compared to Duroc [6,8]. Dietary fat incorporation in 3W pigs may be more centered on other fat depots, such as SC or intermuscular adipose tissue, since this latter showed a higher relative growth rate than Duroc.

The lower rates of d_35_-C18:0 incorporation recorded in Duroc pigs may be explained by the dietary FA dilution with saturated and monounsaturated FA coming from endogenous synthesis and/or desaturation. Several authors have described the increased lipolytic activity of Duroc pigs during their growth [35], and the higher adipogenic and lipogenic gene expression in adipose tissues of fatty pigs [44] in comparison with leaner breeds. Consequently, fat incorporation in Duroc pigs appears to be less dependent on the availability of dietary FA in contrast to the leaner 3W genotype. However, processes associated with lipid mobilization, lipolysis, and extracellular matrix formation are upregulated in lean pigs [22,45,46].

### 4.4. Endogenous Oleic Acid Synthesis: Oleic/Stearic Ratio

Since stearic acid is the main substrate of the SCD enzyme [16], the C18:1 *c*9/C18:0 ratio has been largely used as a measure of apparent SCD activity. In this regard, significant increases in apparent SCD activity with adipose tissue maturity have been reported [33,35]. However, in the present study, apparent FUR was significantly lower in the fattening than in the growing phase; possible reasons will be discussed further below.

Moreover, the authors are unaware of any previous in vivo experimental models using labeled FA as an index of real SCD activity in pigs; the existent bibliography comprises only in vitro models [29,30]. In this approach, (^14^C) oleic acid is obtained through the incubation of tissues with (^14^C) stearic acid, and in line with our results, significant increases in real SCD activity were reported with age in animals between 51 and 95 kg BW [28], and despite this, activity remained unchanged or even decreased up to 128 kg BW. Conversely, previous expression studies described decreases in *SCD* gene expression in adipose tissues as animals fattened, between 60 and 100 kg BW [42]. However, some authors have found no direct correlation between gene expression level and FA profile [47], and these differences could rely on the poorly predicted post-transcriptional regulation of *SCD* among tissues by their mRNA levels [48].

Concerning PT, it is worth mentioning that animals were genotyped for the rs80912566 *SCD* polymorphism, and in the 3W group, five growing and seven fattening pigs were homozygous for the *SCD*_T allele, with the rest (three growing pigs and one fattening pig) being heterozygous (CT). The *SCD*_T allele is almost fixed in some pig breeds, including Landrace and Pietrain, although in Duroc it segregates at intermediate frequencies [15].

Following the present results, both Duroc TT and 3W pigs showed higher apparent and real FUR than Duroc CC pigs, although these differences in real FUR were only found in the fattening phase. Such differences mostly agree with patterns previously reported [15,33], demonstrating that the *SCD*_T allele enhances FA desaturation, with Duroc TT and Duroc CT pigs having 2% and 1% more monounsaturated FA, and 2% and 1% less saturated FA than Duroc CC pigs, respectively [3]. This favorable effect of the *SCD*_T allele has also been confirmed in other Duroc crossbreds such as Duroc × Iberian [15]. Although their pigs were between 95 and 130 kg BW, the authors suggested that the variation in SCD activity was maintained throughout the growing–fattening period.

In our work, the significant decrease of apparent FUR related to animal maturity may be due to a stagnation in the C18:1 *c*9/C18:0 ratio with age, which makes the rate of desaturation per day lower, through (i) lower oleic acid incorporation or (ii) greater stearic acid incorporation into SC. The high availability (8.27 g/kg DM), high digestibility (85.76%), and improved de novo synthesis (245.02%; real FUR) of oleic acid in the diet during the fattening phase suggests that the first possibility (i) should be neglected. In relation to the second possibility (ii), 3W and Duroc genotypes appeared to behave differentially. The increase in the concentration of stearic acid in the SC of fattening 3W pigs is explained by the increase of FIR in this depot during the fattening phase, which also increases the substrate of the SCD enzyme, promoting de novo synthesis [49]. However, the same explanation cannot be applied to the Duroc pigs since FIR remained stable in Duroc TT pigs or slightly decreased in Duroc CC pigs during the growing–fattening period. If exogenous FA incorporation cannot explain the increase in stearic acid pool, then de novo synthesis may account for such increase, as about 80% of total FA deposition comes from biosynthesis [13]. This assumption is also supported by the fact that the SC allometry coefficient in Duroc pigs increased with the same intensity as that of 3W pigs, despite the lower FIR and similar FUR of Duroc pigs compared to 3W pigs in the fattening phase. In that sense, the increased content of unsaturated FA in SC with animal maturity is consistent with previous studies using similar animals [35]. In that case, the reduction in apparent FUR in Duroc pigs is compatible with the increased de novo synthesis of oleic acid, since real FUR accounts for the increase in d_33_-C18:1 *c*9 per unit of d_35_-C18:0; therefore, the incorporation of de novo synthesized unlabeled stearic acid in SC would not alter the d_33_-C18:1 *c*9/d_35_-C18:0 ratio.

## 5. Conclusions

Considering the experimental limitations, stearic and oleic acids have been used to obtain valid indexes of FA incorporation and de novo synthesis, although this latter could only be analyzed in SC. Our findings suggest that fat synthesis in growing pigs is low, and their FA incorporation relies more on direct fat incorporation than on biosynthesis. However, the synthetic FA activity increases significantly with animal maturity, particularly in the fatty Duroc genotypes in which FA accretion relied mostly on the lipogenesis, while the leaner 3W pigs depended more on direct FA incorporation. Therefore, during feed formulation, leaner pigs should have higher dietary FA requirements than fatty ones, while the opposite would be true for FA precursors (i.e., glucose, starch, etc.). In addition, we could find higher biosynthetic action of the *SCD*_T allele of the rs80912566 *SCD* polymorphism.

## Figures and Tables

**Table 1:** Fatty acid composition (% of total fatty acids) of the two-phase experimental diets (growing and fattening), differing in crude protein content (standard, SP vs. low, LP).

Fatty Acids, %	Growing		Fattening	
	LP	SP	LP	SP
Myristic (C14:0)	0.14	0.14	0.26	0.27
Palmitic (C16:0)	14.27	14.17	17.07	17.44
Palmitoleic (C16:1)	0.12	0.12	0.31	0.30
Margaric (C17:0)	0.14	0.15	0.16	0.16
Stearic (C18:0)	2.62	3.06	2.91	3.27
Oleic (C18:1 *c*9)	18.76	18.04	20.14	19.77
Vaccenic (C18:1 *c*11)	0.96	1.00	1.19	1.21
Linoleic (C18:2 *c*9, *c*12)	56.22	55.33	51.73	50.72
Linolenic (C18:3 *c*9, *c*12, *c*15)	5.73	6.96	4.99	5.54
Arachidic (C20:0)	0.33	0.34	0.38	0.39
Eicosenoic (C20:1 *c*11)	0.28	0.27	0.36	0.37
Behenic (C22:0)	0.25	0.28	0.31	0.36
Lignoceric (C24:0)	0.18	0.17	0.21	0.20

**Table 2:** Least square means (± SE) for average daily feed intake (ADFI), average daily gain (ADG), and feed:gain ratio in growing and fattening three-way (3W) crossbred pigs, and purebred Duroc pigs of TT/CC *SCD* genotype.

Items	Producing Type (PT)	Growth Phase (GP)		*p*-Value		
		Growing	Fattening	GP	PT	GP × PT
Animals (*n*)		24	24			
ADFI, g/day	3W	966.84 ± 37.673 ^c^	2131.38 ± 110.492 ^ab^	<0.001	0.159	0.032
	Duroc CC	867.25 ± 37.673 ^c^	2441.48 ± 127.578 ^a^			
	Duroc TT	916.78 ± 37.673 ^c^	2045.23 ± 110.492 ^b^			
Performance data						
ADG, g/day	3W	418.63 ± 31.640 ^cd^	1053.53 ± 99.324 ^a^	<0.001	0.013	0.045
	Duroc CC	440.67 ± 31.640 ^cd^	816.41 ± 106.182 ^ab^			
	Duroc TT	374.37 ± 29.596 ^d^	625.69 ± 106.182 ^bc^			
Feed:gain	3W	2.27 ± 0.215 ^ab^	1.97 ± 0.294 ^b^	0.090	0.228	0.095
	Duroc CC	1.94 ± 0.201 ^b^	2.64 ± 0.314 ^ab^			
	Duroc TT	2.22 ± 0.201 ^ab^	2.96 ± 0.340 ^a^			

^a, b, c, d^ Within each variable, means with different superscripts differ significantly (*p* ≤ 0.05).

**Table 3:** Allometric coefficients (*k*) of liver, loin, ham, and ham components, in two producing types (PTs) of pigs (three-way (3W) crossbred pigs and purebred Duroc pigs) between 80 and 150 days of age with 28 and 87 kg body weight, respectively.

Items	Producing Type (PT)		*p*-Value
	Duroc (*k* ± SE)	3W (*k* ± SE)	PT
Animals (*n*)	20	12	
Whole parts			
Liver	0.73 ± 0.051 *	0.77 ± 0.069 *	0.689
Loin	1.01 ± 0.033	1.03 ± 0.048	0.705
Ham	1.08 ± 0.024 *	1.05 ± 0.027	0.455
Ham components			
Skin and subcutaneous fat	1.37 ± 0.072 *	1.27 ± 0.079 *	0.332
Intermuscular fat	1.02 ± 0.098	1.40 ± 0.111 *	0.015
Intramuscular fat (GM ^1^)	1.42 ± 0.118 *	1.38 ± 0.144 *	0.848
Intramuscular fat (SM ^1^)	0.56 ± 0.062 *	0.76 ± 0.069 *	0.058
Ham muscles	1.08 ± 0.041 *	1.03 ± 0.051	0.345
Biceps femoris	1.11 ± 0.032 *	1.05 ± 0.047	0.884
Gluteus medius	1.09 ± 0.068	1.07 ± 0.081	0.825
Semimembranous	0.81 ± 0.049 *	0.80 ± 0.058 *	0.917
Bones	0.80 ± 0.035 *	0.77 ± 0.047 *	0.666
Sacrum	0.52 ± 0.133 *	0.51 ± 0.150 *	0.955
Coxae	0.85 ± 0.057 *	0.87 ± 0.074	0.828
Femur	0.87 ± 0.041 *	0.82 ± 0.050 *	0.379

^1^ Abbreviations: GM, gluteus medius; SM, semimembranosus. * Allometric coefficients different from 1 (*p* ≤ 0.05).

**Table 4:** Least square means (±SE) for apparent ileal digestibility (AID) of dry matter (DM), crude protein (CP), and ether extract (EE), between three-way (3W) crossbred pigs and purebred Duroc pigs of TT/CC *SCD* genotype, belonging to two growth phases (growing vs. fattening).

AID	Producing Type (PT)	Growth Phase (GP)		*p*-Value		
		Growing	Fattening	GP	PT	GP × PT
Animals (*n*)		24	24			
DM	3W	71.95 ± 2.101 ^c^	78.38 ± 2.667 ^bc^	0.724	0.002	0.053
	Duroc CC	86.52 ± 2.101 ^a^	82.04 ± 2.851 ^ab^			
	Duroc TT	83.82± 2.246 ^ab^	79.66 ± 3.080 ^ab^			
CP	3W	77.59 ± 1.625 ^c^	83.75 ± 2.099 ^ab^	0.547	0.012	0.010
	Duroc CC	88.40 ± 1.737 ^a^	84.56 ± 2.099 ^ab^			
	Duroc TT	84.57 ± 1.737 ^ab^	79.34 ± 2.267 ^bc^			
EE	3W	61.08 ± 3.936 ^c^	82.25 ± 2.778 ^ab^	< 0.001	0.011	0.045
	Duroc CC	80.59 ± 3.936 ^ab^	84.24 ± 2.970 ^a^			
	Duroc TT	74.10 ± 3.936 ^b^	83.49 ± 3.208 ^ab^			

^a, b, c^ Within each nutrient, means with different superscripts differ significantly (*p ≤* 0.05).

**Table 5:** Least square means (±SE) for fractional incorporation rate (FIR, %) of deuterated stearic acid (d_35_-C18:0) in the liver, subcutaneous adipose tissue (SC), longissimus dorsi (LD) and semimembranosus (SM) muscles, between three-way (3W) crossbred pigs and purebred Duroc TT/CC for the *SCD* genotype, belonging to two growth phases (growing vs. fattening).

FIR	Producing Type (PT)	Growth Phase (GP)		*p-*Value		
		Growing	Fattening	GP	PT	GP × PT
Animals (*n*)		24	24			
LIVER	3W	11.87 ± 1.746 ^b^	52.50 ± 6.005 ^a^	<0.001	0.242	0.139
	Duroc CC	13.61 ± 1.746 ^b^	37.95 ± 6.487 ^a^			
	Duroc TT	12.69 ± 1.746 ^b^	37.98 ± 6.005 ^a^			
SC	3W	18.90 ± 2.717 ^b^	31.76 ± 3.876 ^a^	0.415	<0.001	0.037
	Duroc CC	11.78 ± 2.717 ^bc^	7.38 ± 4.475 ^c^			
	Duroc TT	13.62 ± 2.905 ^bc^	12.22 ± 3.876 ^bc^			
LD	3W	14.45 ± 1.618 ^a^	4.31 ± 0.464 ^b^	<0.001	<0.001	0.051
	Duroc CC	7.54 ± 1.618 ^b^	1.57 ± 0.536 ^c^			
	Duroc TT	6.62 ± 1.618 ^b^	2.33 ± 0.464 ^c^			
SM	3W	1.39 ± 0.176 ^a^	Under LOQ *	0.019	0.187	0.040
	Duroc CC	1.31 ± 0.176 ^a^	0.63 ± 0.120 ^b^			
	Duroc TT	1.26 ± 0.176 ^a^	1.22 ± 0.111 ^a^			

^a, b, c^ Within each tissue, means with different superscripts differ significantly (*p* ≤ 0.05). * Value under the limit of quantification.

**Table 6:** Least square means (± SE) for apparent and real fractional unsaturated rate (FUR; %/day) in subcutaneous adipose tissue between three-way (3W) crossbred pigs and purebred Duroc of TT/CC *SCD* genotype, belonging to two growth phases (growing vs. fattening).

FUR	Producing Type (PT)	Phase (GP)		*p*-Value		
		Growing	Fattening	GP	PT	GP × PT
Animals (*n*)		24	24			
Apparent	3W	5.47 ± 0.231 ^a^	2.03 ± 0.121 ^d^	<0.001	<0.001	<0.001
	Duroc CC	2.84 ± 0.216 ^c^	1.60 ± 0.121 ^e^			
	Duroc TT	4.25 ± 0.216 ^b^	1.99 ± 0.121 ^d^			
Real	3W	2.16 ±0.300 ^c^	7.23 ± 0.617 ^a^	<0.001	0.107	0.016
	Duroc CC	2.14 ± 0.300 ^c^	5.28 ± 0.660 ^b^			
	Duroc TT	1.56 ± 0.300 ^c^	7.68 ± 0.617 ^a^			

^a, b, c, d, e^ Within each rate, means with different superscripts differ significantly (*p* ≤ 0.05).

## Data Availability

The data presented in this study are available on request from the corresponding authors.
